# A Highly Energy-Efficient Body-Coupled Transceiver Employing a Power-on-Demand Amplifier

**DOI:** 10.34133/cbsystems.0030

**Published:** 2023-08-08

**Authors:** Tao He, Yabin Zheng, Xu Liang, Jiamin Li, Longyang Lin, Wenfeng Zhao, Yongfu Li, Jian Zhao

**Affiliations:** ^1^ Department of Micro/Nano Electronics, Shanghai Jiao Tong University, Shanghai, China.; ^2^ School of Microelectronics, Southern University of Science and Technology, Shenzhen, China.; ^3^ Department of Electrical and Computer Engineering, Binghamton University, Vestal, NY, USA.

## Abstract

Wearable body sensor nodes require massive data transmission under limited energy. However, it suffers from drastically varying channel loss, which limits its energy efficiency in practical scenarios. This paper presents a power-driven body-channel transceiver (TRX), whose power consumption can be adaptively tuned against varying channel loss. An out-band programmable gain amplifier (PGA) is proposed to save power and generate a quasi-linear correlation between PGA gain and power. By using the quasi-linear gain-power relationship, we propose an auto gain/power control technique to realize on-demand power consumption. In addition, a differential balanced transmitter is designed to eliminate base-band harmonics in on-off keying modulation and increase the power delivered by the transmitter (TX). The TX and receiver (RX) of the prototype were integrated into 1 chip and fabricated in a 55-nm complementary metal oxide semiconductor process. During the measurement, 2 chips were configured as TX and RX, respectively. Both the TX and the RX were wearable, powered by lithium batteries, and attached to the subject’s hands. The prototype achieved a 5.25-Mbps data rate with 16-pJ/bit energy efficiency at a 1.5-m straight-line ground path distance. Furthermore, the proposed TRX maintained stable communication within a 1.5-m distance, while dynamically reducing power consumption.

## Introduction

Wireless body area network (WBAN) is an emerging technique that offers distributed, real-time, long-term, and low-cost vital sign monitoring [1,2]. It has the potential to be employed in medical diagnosis or human–machine interfaces [3]. Massive amounts of data need to be communicated throughout the body as the scale of WBAN increases. Conventional radio frequency communication methods like Bluetooth and Zigbee suffer from the human body shielding effect [4], which results in high power consumption and limits the node lifetime.

Body-coupled communication (BCC) utilizes the human body as the transmission medium, which has a lower path loss that improves the transmission power efficiency by around 2 orders of magnitude [5,6]. Therefore, it is considered one of the most promising candidates in WBAN. BCC has already been successfully implemented in state-of-the-art works such as hearing aids [7], capsule endoscopes [8], concurrent electroencephalography recording [9], power transmission [10], and authentication [11].

**Fig. 1.**
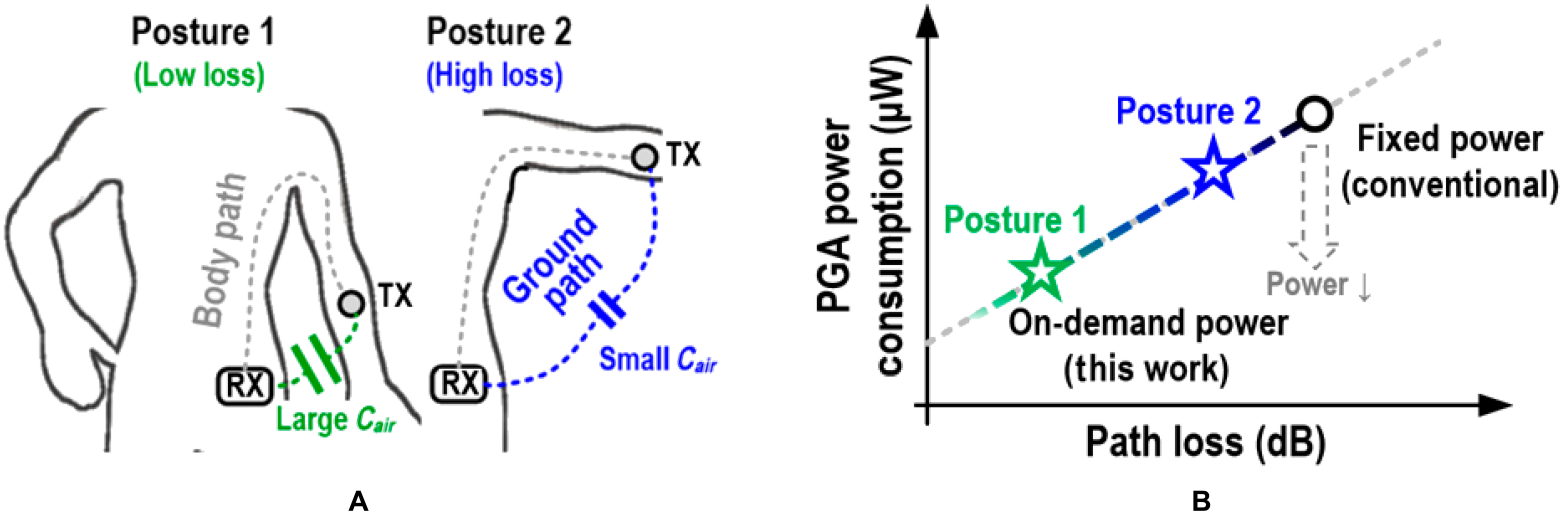
Illustration of the power-on-demand TRX. (A) The ground path is related to posture, which causes a varying body channel problem. (B) The proposed power-on-demand amplifier realizes a quasi-linear gain-power relationship, whose state is calibrated by the AGPC technique to realize high energy efficiency under variable channel conditions.

As BCC is extended to cover a wider range of body parts, the varying body channel will be an issue. As shown in Fig. 1A, the BCC transmission channel consists of human tissue (body path) and the coupling capacitance *C_air_* between the ground electrodes of the transmitter (TX) and receiver (RX) (ground path). In practice, the ground path can introduce an up-to-40-dB loss and become the bottleneck of the communication channel [12,13]. Additionally, since *C_air_* is related to the TX–RX distance, as illustrated in Fig. 1A, the change of posture introduces a disturbance mainly in the ground path. A 10-dB channel gain disturbance is measured in this work as the TX–RX distance ranges from 5 to 150 cm. To overcome the ground path problem, an off-chip inductor is inserted into the channel to resonate with the ground capacitance and compensate for the channel loss [14]. Moreover, the inductor can be tunable [15] or self-adaptive [16] to tackle the varying channel loss. However, as the input impedance is in series with the compensation inductor, a low value (usually less than 50 Ω) is recommended to prevent degradation of the quality factor. In contrast, the optimal input impedance typically exceeds 500 Ω. Therefore, the inductor-based compensation scheme faces a dilemma [17,18]. The channel loss from varying backward paths is still not effectively compensated.

This paper presents an inductor-free energy-driven body-channel transceiver (TRX), whose power consumption can be adaptively tuned against varying channel loss. Instead of implementing a resonance inductor, an out-band programmable gain amplifier (PGA) and an auto gain/power control (AGPC) are proposed to counteract the unstable body channel without bulky inductors. As shown in Fig. 1B, the proposed RX achieves a quasi-linear gain-power relationship by utilizing the out-band PGA. Since the demanded RX gain and the ground path distance have a positive correlation, as the channel distance changes, the AGPC controls the RX power to optimize the operating state and dynamically saves power. Hence, the AGPC realizes on-demand power delivery corresponding to the ground path distance.

Conventional BCC designs regard the TX ground electrode (E_GND−TX_) as the TX circuit ground, which introduces a DC component (a base-band harmonic of the modulated data spectrum) in the TX-injected signal and degrades the TX energy efficiency and maximum data rate. By utilizing the E_GND−TX_ as an output terminal, rather than the circuit ground, a differential balanced transmitter (DBT) is proposed. The differential signalling extends the TX-injected signal range from 0∼VDD to −VDD∼VDD, doubling the TX signal swing to maintain communication in the poorest channel case. Furthermore, DBT eliminates the base-band harmonic, which increases data rate and TX efficiency.

The rest of this paper is organized as follows: Materials and Methods presents the motivation and system design consideration of the proposed BCC TRX with AGPC and DBT. Measurement results are present in Results, followed by Conclusion.

## Materials and Methods

**Fig. 2.**
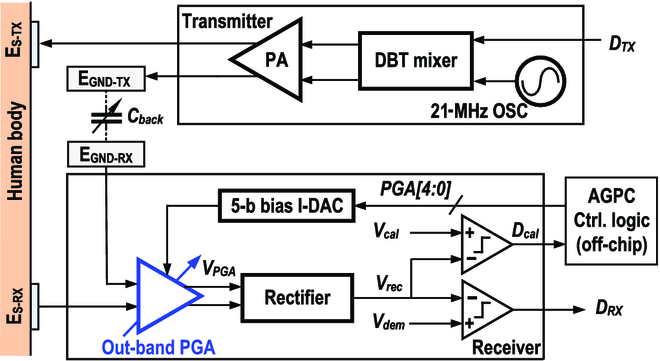
The overall architecture of the proposed BCC TRX.

Figure 2 depicts the system block design of the proposed BCC TRX. In the TX, the DBT mixer modulates the base-band TX data (*D_TX_*) on the 21-MHz carrier provided by a digital-controlled oscillator. There is a 1-cycle phase shift between the dual outputs of DBT mixer to generate a tristate modulated on-off keying (OOK) signal, which eliminates the base-band harmonic while boosting the signal swing by 2×. Thus, the maximum data rate and maximum TX–RX distance are enhanced.

The modulated differential digital signal is transmitted to the human body through the TX signal electrode (E_S−TX_) and injected into the TX ground electrode (E_GND−TX_) by a digital power amplifier. In the RX, the PGA is specially designed to realize a direct quasi-linear trade-off between power and gain, by amplifying the OOK signal whose carrier frequency is higher than the PGA’s 3-dB bandwidth. AGPC system automatically calibrates power gain according to channel loss to save power. The subsequent rectifier demodulates the OOK signal. Its output is compared to *V_dem_* to generate the demodulated base band *D_TX_*, while it is also compared to *V_cal_* to evaluate and calibrate the state of the RX. The *D_cal_* signal is an indicator of the PGA output signal strength. If the PGA output strength is larger than the signal-to-noise ratio requirement of demodulation, the 5-bit PGA bias current digital-to-analog converter (DAC) is calibrated to decrease the PGA power to a proper state, thus dynamically saving the power consumption.

### The out-band PGA

**Fig. 3.**
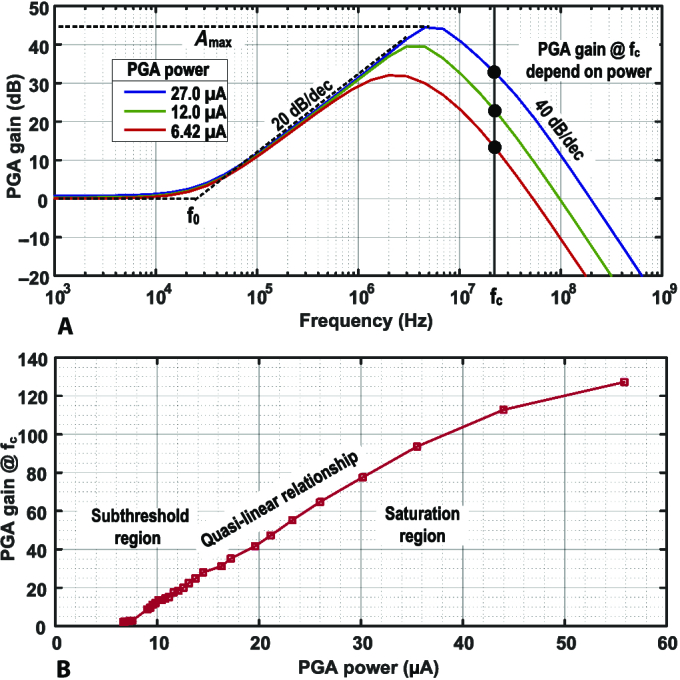
The gain behavior of the proposed out-band PGA. (A) The frequency response. The gain at 21 MHz is correlated with power. (B) The measured PGA gain at 21 MHz corresponding to the power with a quasi-linear relationship.

Conventional PGAs amplify signals within their 3-dB bandwidth, where their gain-bandwidth product (GBW) is required to cover the worst-case scenario, and power can be extremely high when a channel has a high loss. To dynamically save power according to channel conditions, an intuitive approach is to make the 3-dB bandwidth stay constant and always track the signal bandwidth under the variation of the gain, so that the GBW is always kept at a minimum level. However, this involves the detection and optimization of both GBW and the feedback factor, which is complicated to implement. To achieve auto power saving and optimize the approach, the out-band PGA is proposed by restricting the 3-dB bandwidth below the narrow-band OOK signal bandwidth and amplifying at the rolling-off band, or out-band. As an open-loop amplifier in the out-band region, the PGA reduces power consumption since there is no stability issue compared to close-loop counterparts. There is no need for additional frequency compensation, which trades bandwidth for stability. Hence, the out-band PGA exhibits low-power characteristics. Additionally, as illustrated in Fig. 3, the PGA gain and power are designed to exhibit a quasi-linear correlation, and the AGPC can be realized by tuning the tail current only.

As a result of the out-band operation, processes, voltages, and temperatures could impair the RX. Hence, special care should be taken for the design margin. Considering the 40-dB channel loss [12,13] and the RX sensitivity, the PGA needs to provide an up to 30-dB gain under processes, voltages, and temperatures variation. That is achieved by designing a maximum 45-dB PGA gain in the typical case, along with a wide-ranged tail current (the 5-bit current DAC), whose reference voltage can be separately provided to further extend the PGA tail current.

**Fig. 4.**
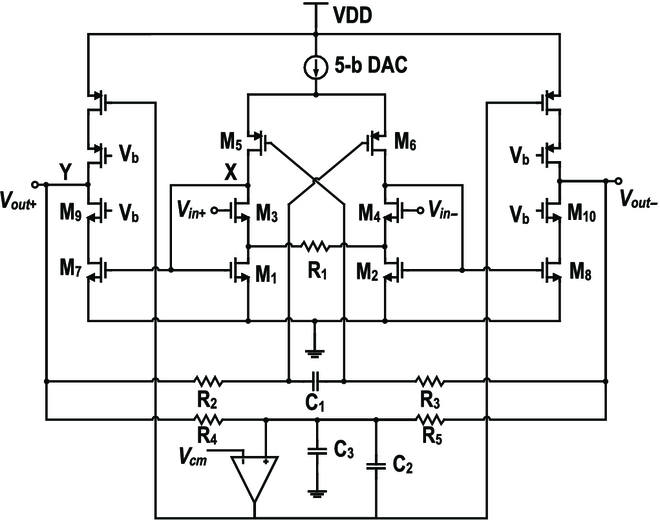
Schematic of the proposed out-band PGA.

Figure 4 depicts the out-band PGA design. The PGA utilizes a flipped voltage follower structure [19] for the input pair. With the local feedback provided by the flipped voltage follower structure, the current drawing through *M*_3, 4_ is kept constant. This improves the linearity of the source follower, which copies *V_in_* to *R*_1_, generating a current signal *V_in_*/*R*_1_. The second stage is a cascode current buffer, which mirrors *V_in_*/*R*_1_ to the output nodes, where there are 2 branches designed as load. The PGA gain is determined by the dominance of *R*_4_ and *R*_5_ on the load branches (*R*_4, 5_ ≪ *R*_2, 3_). Furthermore, *R*_4, 5_ and *C*_3_ are implemented as a common mode feedback sensor, with an inserted amplifier and miller capacitor *C*_2_, and the output common mode is converged to *V_cm_*. *C*_3_ is designed for fast start-up. *R*_2, 3_ and *C*_1_ is a passive low-pass feedback branch, which reduces the PGA to a unity-gain buffer at low frequencies to suppress the DC input offset [20] and the low-frequency noise like power-line interference.

**Fig. 5.**
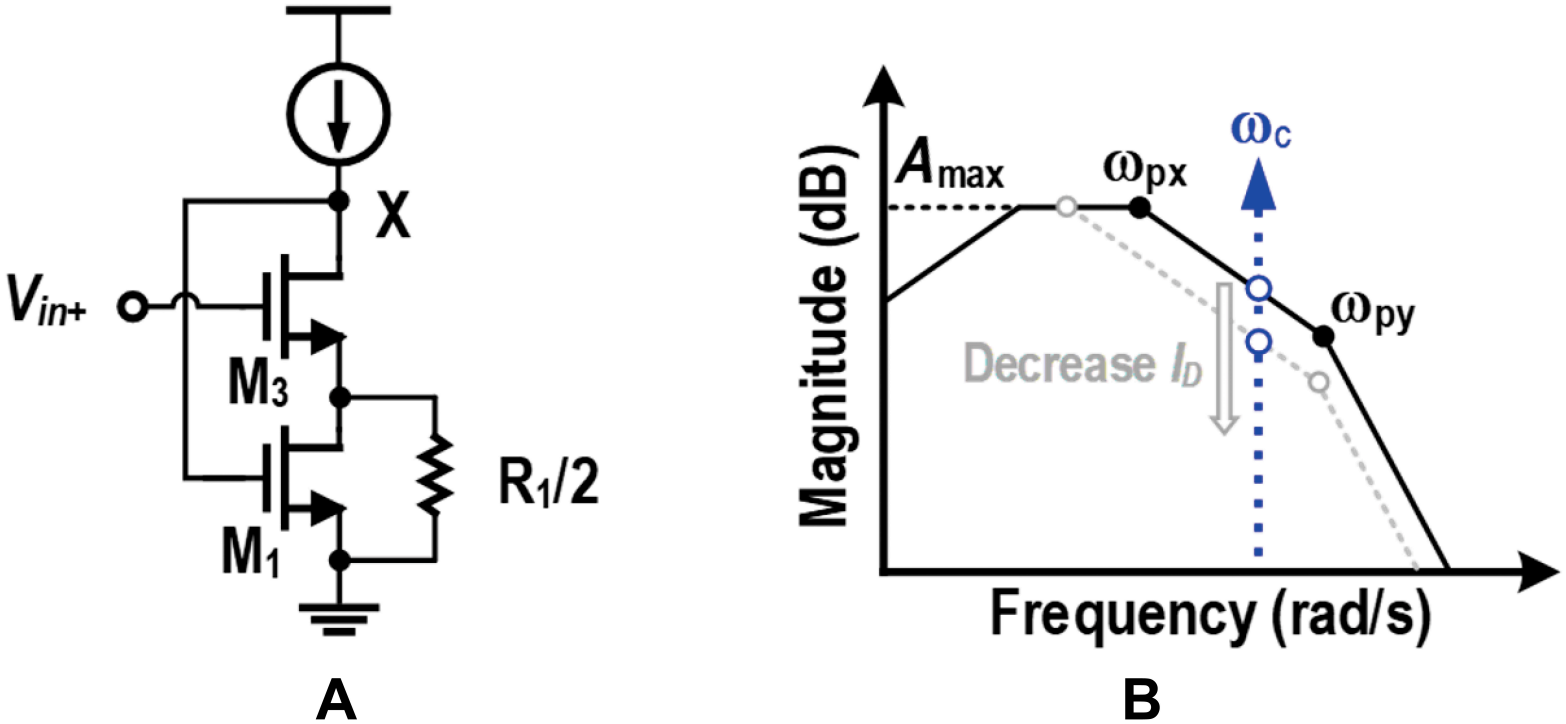
Frequency response analysis of the proposed out-band PGA. (A) Simplified small-signal half-circuit model to calculate node resistance at node X, and (B) frequency response and pole design for the out-band PGA to achieve quasi-linear power-driven operation.

The typical frequency response of the proposed out-band PGA is illustrated in Fig. 5B, where *f_c_* is the 21-MHz OOK carrier frequency. The dominant poles are located at nodes X (*P_X_*) and Y (*P_Y_*). To realize a power-driven programmable gain, the modulated narrow-band signal, centered at *f_c_*, should be designed between *P_X_* and *P_Y_*. It is vital to analyze how *P_X_* and *P_Y_* are located for maintaining enough margin. To calculate *P_X_* the simplified half-circuit model shown in Fig. 5A can be adopted. A test voltage at node X excites a current *g*_*m*1_*V_test_*, which is divided into 2 branches with a resistance of 1/*g*_*m*3_ and *R_1_/2*, respectively. The current flowing through *M*_3_ becomes the test current *I_test_*, which yields the node resistance *R_X_* by *V_test_*/*I_test_*, illustrated as Eq. 1 (*R*_1_ = 300 Ω and 1/*g*_*m*3_ ≫ *R*_1_). *P_X_* (Eq. 2) is designed as the dominant pole by designing a small *R*_1_. Since *P_X_* is linearly correlated to *I_D_*, it follows the trend of tail current and realizes a quasi-linear power-driven gain, while *P_Y_* is determined by the load *R*_4,5_ and the parasitic capacitor *C_Y_*, as shown in Eq. 3. To avoid degrading the available gain, *R*_4,5_ is designed to push *P_Y_* above *f_c_*.

$$R_{X}=\frac{R_{1}/2+1/g_{m3}}{R_{1}/2}\frac{1}{g_{m1}}=\frac{2}{g_{m1}g_{m3}R_{1}}$$ (1)

$$P_{X}=\frac{1}{2\pi R_{X}C_{X}}=\frac{\mu_{n}C_{\mathrm{ox}}\sqrt{{\left(W/L\right)}_{1}{\left(W/L\right)}_{3}}R_{1}I_{D}}{2\pi C_{X}}$$ (2)

$$P_{Y}=\frac{1}{2\pi R_{4}C_{Y}}$$ (3)

The out-band PGA achieves programmable gain with a fixed feedback factor, yielding a maximum available gain *A_max_* that should suffice in the worst case, shown as Eq. 4, while *f*_0_ is determined by the passive low-pass feedback filter *R*_2,3_ and *C*_1_, shown as Eq. 5. *f*_0_ should be low enough to reveal *A_max_* before *P_X_*.

$$A_{\max}=\frac{{\left(W/L\right)}_{7}\left(R_{4}+R_{5}\right)}{{\left(W/L\right)}_{1}R_{1}}$$ (4)

$$f_{0}=\frac{1}{2\pi \left(R_{2}+R_{3}\right)C_{1}}$$ (5)

Following the design considerations described above, the proposed out-band PGA achieves quasi-linear power-driven programmable gain. As illustrated in Fig. 3A, the simulated frequency responses exhibit various dominant pole allocations under different power consumption. The measured gain at *f_c_* is shown in Fig. 3B; by applying a 5-bit bias current DAC, the proposed out-band PGA achieves a 0 to 120 gain-tuning range at the carrier frequency under a power consumption of 6 to 56 μA. Moreover, the gain-power relationship exhibits quasi-linear behavior in the saturation and subthreshold regions.

### The AGPC

The out-band PGA enables power-driven operation. The AGPC logic will stabilize the amplitude of the amplified received signal against varying channel loss by dynamically tuning the PGA’s power.

**Fig. 6.**
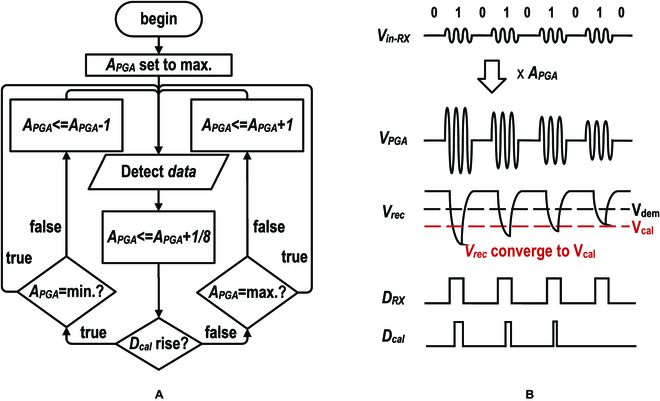
Illustration of the AGPC. (A) Flowchart of the AGPC algorithm and (B) time domain illustration of the AGPC calibration.

Figure 6A shows the flowchart of the APGC system. The gain/power of the PGA is initially set to the maximum value. The system will keep monitoring the amplitude of the rectifier output (*V_rec_*) and comparing it with a preset reference *V_cal_*. If the *V_rec_* < *V_cal_*, the omparison result (*D_cal_*) will be active to increment the *A_PGA_*, and vice versa. Finally, the amplitude will converge to *V_cal_*. Figure 6B shows a typical waveform of the proposed AGPC system.

In AGPC, robustness is considered through *V_dem_* and *V_cal_*. A lower *V_dem_* enhances the tolerance for noise and interference at the expense of a higher *V_rec_* swing, which indicates higher power. The difference between *V_dem_* and *V_cal_* determines the signal-to-noise ratio of demodulation. Furthermore, *V_dem_* − *V_cal_* should be large enough to prevent *V_rec_* swing drops above *V_dem_* as the ground path distance increases, which causes system failure. As human movement is extremely slow compared to AGPC calibration, system failure occurs only when TRX is abruptly loosen. It is thus possible to assign a periodic rise of the PGA gain to the recovery effort.

### The DBT design

Conventional BCC utilizes the E_GND−TX_ as the TX circuit ground. Consequently, a base-band signal component remains at the output after OOK modulation. The emitted low-frequency harmonic attenuates the narrow-band OOK signal and limits the data rate. Although the low-frequency gain truncation of PGA enhances the tolerance to base-band interference, it is difficult to eliminate because the maximum base-band signal bandwidth is on the same order of magnitude as the OOK carrier frequency.

**Fig. 7.**
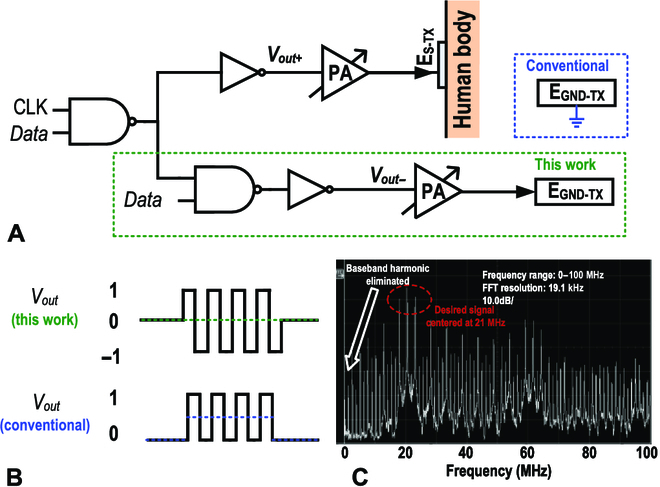
Design and properties of the proposed DBT. (A) The DBT design. (B) The DBT eliminates base-band harmonic while doubling the signal swing. (C) The spectrum of DBT output. FFT, fast Fourier transform.

As illustrated in Fig. 7, the proposed DBT adopts both E_S−TX_ and E_GND−TX_ as output terminals, while the TX circuit ground is excluded from the BCC signal path. By applying a phase-shifted OOK modulated signal at E_GND−TX_, the differential TX output is able to cancel the base-band harmonic of TX OOK output spectrum and increase the maximum TX signal swing by 2× under the limited supply voltage. Figure 7B illustrates the difference of *V*_*out*+_ and *V*_*out*−_ (*V_out_*) is a tristate OOK modulated signal with doubled swing and no DC component. Figure 7C depicts the fast Fourier transform of DBT output that shows its spectrum. The base-band signal is modulated on the odd harmonics of the carrier clock, while the remaining high-order harmonics can be suppressed by the PGA and off-chip passive filters.

## Results

**Fig. 8.**
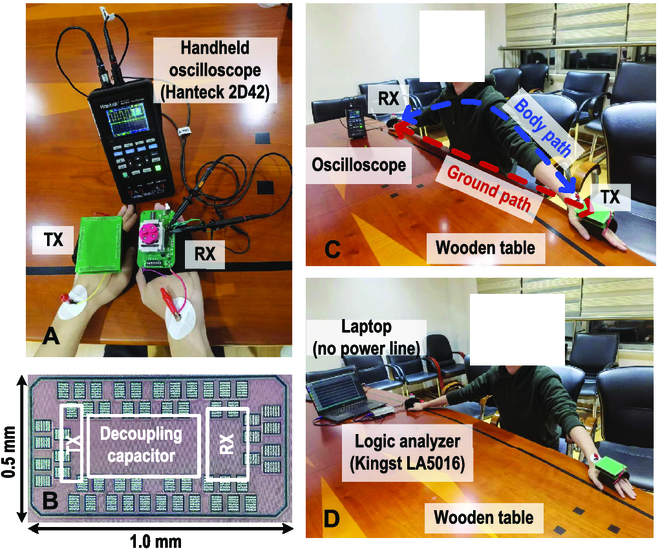
Measurement setup. (A) TX and RX attached on hands, with a handheld oscilloscope to capture the waveform. (B) Chip photograph. (C) Handheld setup to measure *V_PGA_*, *D_RX_*, and *D_cal_*, (D) setup to record *D_RX_* and measure BER.

**Fig. 9.**
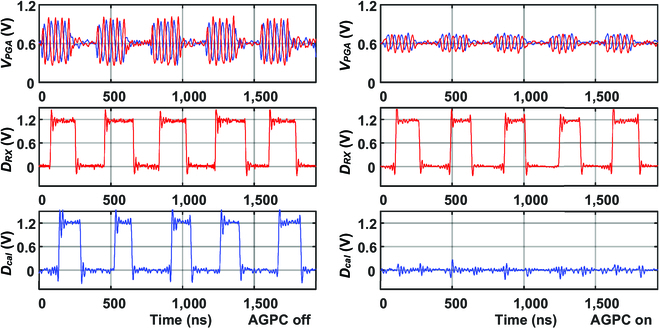
Measured steady-state *V_PGA_* (differential), *D_RX_*, and *D_cal_* waveform with and without AGPC, while transmitting a 5.25-Mbps OOK signal.

**Fig. 10.**
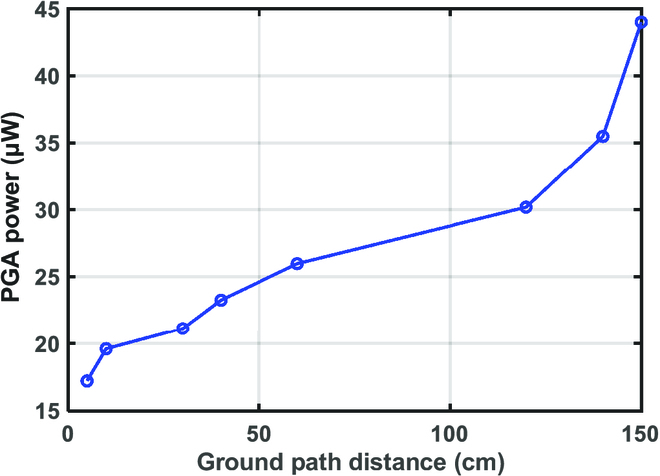
Measured PGA power versus ground path distance.

The proposed auto-power/gain controlled RX and differential balanced TX were fabricated in a 55-nm complementary metal oxide semiconductor process. Microphotographs of the die and measurement setup are shown in Fig. 8. As part of the DBT implementation, the E_GND−RX_ is designed as a box to shield the RX circuit ground and the battery. Both the TX and RX are implemented as wearable devices and attached to the hands, with a handheld oscilloscope (Hanteck 2D42) to measure and capture the waveform. During the measurement, as Fig. 8C illustrates, TX and RX are attached to both hands, and the straight-line distance is recorded as the ground path distance. As the subject’s 2 hands get closer, the ground path distance decreases while the body path distance remains the same. Figure 9 shows the recorded steady-state waveform with AGCP off and on, while transmitting a 5.25-Mbps OOK signal. When AGPC is off, the PGA is initialized in a high-gain state. The amplified OOK signal at the PGA output (*V_PGA_*) has a large swing that generates demodulated base-band data (*D_RX_*) and triggers the calibration indicator signal (*D_cal_*). As AGPC is on, AGPC downgrades the PGA gain according to *D_cal_* to save power dynamically. Eventually, the PGA gain and power converge to the optimized state where *V_PGA_* becomes smaller so that *D_cal_* signal is approaching the edge to rise. During waveform capturing and data recording, the handheld oscilloscope or a logic analyzer (Kingst LA5016) is connected to a battery-powered laptop (as in Fig. 8D). It is noted that as the measurement devices are connected to the laptop, the ground path is strengthened by its large volume and the received signal becomes stronger. Hence, we adjusted the PGA gain to make the waveform resemble that without connecting to the laptop. Moreover, the noise level increased substantially with the laptop connected, likely due to the increased reception of AM radio. The measured PGA power versus ground path distance is depicted in Fig. 10, the PGA power is saved up to 60% according to the ground path distance. The performance comparison with previously reported narrow-band BCC TRXs is shown in Table; this work achieved a high RX energy efficiency of 16 pJ/bit at a 1.5-m ground path distance.

**Table.** Performance comparison with the previously reported BCC TRXs.

Parameter\Pub.	JSSC’ 17 [6]	ASSCC’ 19 [18]	JSSC’ 19 [21]	JSSC’ 20 [22]	ESSCIRC’ 22 [23]	This work
Process (nm)	65	180	65	180	28	55
Supply (V)	1.1	0.8	1.2	1.5	TX:1.2 RX:1	1.2
Data rate (Mbps)	0.2–2	4	1.3125	105	30–50	5.25
Modulation	P-OFDM	OOK	FSDT	QPSK	NRZ	OOK
TX power (μW)	870	120	3,520	2,100	1,686	624
RX power (μW)	1,100	160	620	9,400	3,442	84 ^a^
RX efficiency (pJ/bit)	550	41	472	90	68.84	16 ^a^
Bit err. rate	<10^−7^	<10^−5^	<10^−7^	<2 × 10^−4^	<1.5 × 10^−4^
AGPC	No	No	No	No	No	Yes

^**a**^ Measured at 1.5-m ground path distance

## Conclusion

A power-on-demand BCC TRX with an out-band PGA and DBT is proposed in this paper. As a result of the out-band design, the RX is low-power and exhibits a quasi-linear gain-power relationship. With the AGPC, the varying body channels can be accommodated by auto calibration. The DBT increases the transmission distance and enhances energy efficiency on the TX side. According to in vivo measurements, the prototype maintains stable communication at a data rate of 5.25 Mbps within a distance of 1.5 m on the human body. With the AGPC on, the TRX dynamically saves up-to-60% PGA power when the TX–RX ground path distance changes. Thanks to the low-power design, this work achieves a high RX energy efficiency of 16 pJ/bit at a 1.5-m ground path distance. Further, this work dynamically saves power as the posture of the human changes, which extends the capabilities of BCC for further applications.

## Data Availability

The data are freely available upon request.
